# Determination of Optimal Line-Heating Conditions for Flatness Control of Wind Tower Blocks Using Strain as Direct Boundary Method

**DOI:** 10.3390/ma15227962

**Published:** 2022-11-10

**Authors:** Hee-Chan Yoon, Hun-Bong Lim, Hong-Jun Noh, Young-Hwan Han, Jae-Chul Lee, Hyun-Ik Yang

**Affiliations:** 1 Department of Mechanical Design Engineering, Hanyang University, 222, Wangsimni-ro, Seongdong-gu, Seoul 04763, Korea; 2 Department of Mechanical Design Engineering, Myongji College, 134, Gajwa-ro, Seodaemun-gu, Seoul 03656, Korea; 3 Material & Component Convergence R&D Department, Korea Institute of Industrial Technology (KITECH), Hanggaul-ro 143, Sangnok-gu, Ansan-si 15588, Korea

**Keywords:** flatness, tilt angle, line heating, strain as direct boundary

## Abstract

The wind tower block is welded with the flange to assemble the wind tower. The inherent strain due to local heating and cooling of the weld affects the flatness of the flange. Therefore, line heating is performed to satisfy the design criteria of the flange flatness, but the work variables depend on the operator’s empirical judgment. This study proposed a method to determine the optimum linear heating conditions to control the welded flatness of wind tower blocks and flanges. A proposed method uses the inherent strain method, a simple analysis method, and the optimization is performed based on the deformation superposition method. The changes in flange flatness due to welding and single-point heating were calculated. Then, the flatness change due to single-point heating is superimposed with a scale factor, which represents the magnitude of line heating, and is added to the flatness change due to welding. Using the optimization procedure, the line heating conditions used to derive the flatness that satisfies the design criteria were derived and applied to the analytical model for verification.

## 1. Introduction

The rapid heating and cooling process of welding causes internal expansion and shrinkage of the weld, and residual deformation is generated after welding is completed. Residual deformation reduces the strength of the structure and affects the assembly process according to the tolerance; therefore, a post-treatment process is required to remove it. Line heating is a procedure to remove out-of-plane deformation by generating a symmetrical residual deformation based on a neutral plane through a heating and cooling process using a heat input source such as a torch and induction. In addition, line heating is used to remove the deformation and process it into the desired shape. Therefore, experiments and numerical analysis are being conducted to investigate the relationship between the factors affecting line heating and deformation.

Fujishiro et al. [1] performed a sensitivity analysis on the variables affecting line heating using an experimental method. The analysis was performed on seven variables using an orthogonal array table, and the plate thickness showed the most significant sensitivity. Satoh et al. [2] studied the angular deformation during heating and cooling and found that most of the deformation occurred during cooling. Lim et al. [3] investigated the changes in the physical properties due to the line heating and cooling process. They confirmed that the grains in the heat-affected zone (HAZ) were refined as ferrite and pearlite. Nomoto et al. [4] analyzed the deformation caused by line heating by dividing the cooling region of the water-cooled process, considering the actual cooling rate and volume percentage of each phase in the inherent strain region after line heating. Hwang et al. [5] analyzed the thermal deformation of a curved plate due to line heating using the shape of the HAZ derived from a flame-bending experiment.

Since the development of numerical analysis, many studies have been conducted. Lee et al. [6] proposed a method for predicting plate deformation by organizing the variables for line heating using multivariate analysis. Nomoto et al. [7] developed a deformation analysis method by converting the angular deformation and shrinkage due to line heating into equivalent loads and applying them to a finite-element model. Ko et al. [8] derived the deformation due to line heating by defining the inherent strain for the HAZ to calculate the equivalent load. Ha [9,10,11] developed a strain as direct boundary (SDB) method that applies inherent strain as an equivalent temperature boundary condition and predicts the thermal deformation of unit specimens and large structures. Noh et al. [12] proposed a method of installing permanent stiffeners such that the effect of line heating does not disappear during the lifting process of the ship. The properties affected by the phase transformation were calculated considering the properties and fractions of each phase for the heating and cooling of line heating. By applying this, the efficiency of line heating as analyzed according to the shape and distance of the stiffeners.

Wind tower blocks are welded with flanges for assembly with other blocks. Residual deformation due to the welding affects the flatness of the flange. Zhang et al. [13] investigated residual stress distribution and evolution in single-pass bead-on-plate TIG welding of S355J2 steels using a thermo-metallurgical–mechanical coupling model. The prediction result of the welding residual stress confirmed that the solid-state phase transformation incorporating undercooling austenite and transformation strain is an important factor influencing the welding residual stress. Hensel et al. [14] compared fatigue tests for welded joints, including the microstructure, hardness, phase transformation temperature, and mechanical properties of base materials S355J2 and S960QL using low transformation temperature (LTT) filler and conventional filler. As a result, it was confirmed that the Fe-Cr-Ni-based LTT-metal cored filler material can be reliably welded without cracking or pore formation. Heinze et al. [15] revised the numerical model considering residual stress evolution in the heat-affected zone to welding of low-alloy structural steel S355J2 and heat-resistant steel P91. For structural steel S355J2, no significant influence on the residual stress state due to the usage of the enhanced model was found. For P91, which transforms to become fully martensitic, the residual stress calculation appears to be a difference of up to 200 MPa. It was confirmed that the simulation quality of the material exhibiting transforms fully martensitic can be improved by considering the Ms-temperature as a function of pAGS.

In the wind tower assembly process, the flatness of the flange must satisfy the design criteria. Line heating is performed on-site to satisfy the design criteria for flatness; however, the operator’s empirical judgment determines the work variable. Therefore, this study proposes a method of determining optimal line heating conditions to satisfy the design criteria for the flatness change generated by welding the wind tower block and flange. The temperature boundary conditions for the multi-layer butt welding of the wind tower blocks and flanges were obtained using the SDB method proposed by Ha [9]. Subsequently, the temperature boundary conditions were applied to the three-dimensional shell model to obtain the change in the flatness and tilt angle of the flange by welding. In addition, the change in the flatness and tilt angle of the flange was obtained when line heating was performed at a single point. The flatness change for a single point was superimposed in the line-heating direction to offset the flatness change due to welding. At this time, the flatness change due to single-point heating is superimposed with a scale factor, which means the magnitude of line heating, and is added to the flatness change due to welding. The optimal scale factor satisfying the design criteria for the flatness and tilt angle of the flange was obtained using optimization.

## 2. Procedures to Determine Optimal Line Heating Condition

**Figure 1:**
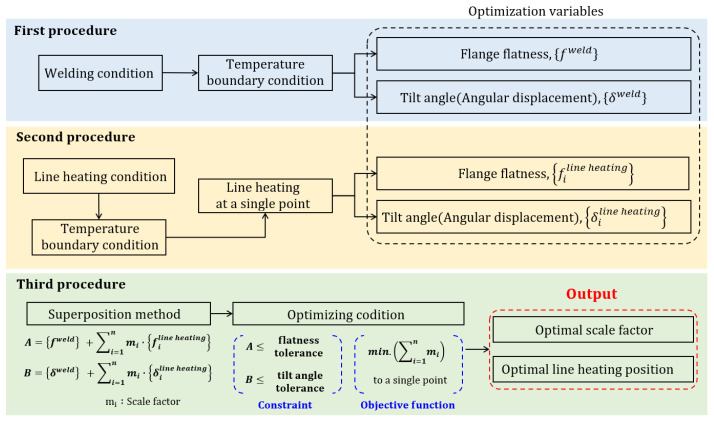
Procedures for determining the line-heating condition.

The process of determining the optimal conditions for line heating to offset the deformation due to welding is divided into three procedures, as shown in Figure 1. This section briefly describes each of these procedures. The first procedure calculates the flatness and tilt angle of the flange due to welding the wind tower block and flange. To perform the calculation, the temperature boundary condition of x-groove multilayer butt welding was obtained using the SDB method. Subsequently, the flatness and tilt angle were calculated from the difference in the axial direction displacement of the inner and outer edges of the flange. The second procedure involves calculating the flatness and tilt angle of the flange due to line heating. When line heating is performed on a constant area, the heat-affected zone (HAZ) shape is an equilateral trapezoid. Therefore, the temperature boundary condition can be derived using the SDB method. Line heating was performed at a single point between the inner and outer edges of the flange, and at all single points between the edges to derive the heating position. The flatness and tilt angle due to line heating at a single point were calculated in the same manner as in the first procedure. The third procedure formulates an optimization function based on the deformation superposition theory to calculate the scale factor and line-heating position where the flatness and tilt angle of the weld are offset. The input variables for this calculation are the flatness and tilt angle of the flange due to welding and single-point heating. The scale factor is calculated by superposing with the flatness and tilt angle of the line heating and summing the weld deformation in the direction of the line heating. For each procedure, the single point that satisfies the design criteria and where the sum of the scale factors is the minimum is determined as the line heating position.

## 3. Flange Tolerance Requirements

Since 2000, there has been a problem with the connecting bolts of the tower block and the flange breaking during the wind tower operation. The leading cause of this problem was identified as the manufacturing and installation tolerance of the flanges. Manufacturing and installation tolerances are divided by the flatness and tilt angle of the flange top, which have a decisive effect on the structural integrity after bolting [16,17].

**Figure 2:**
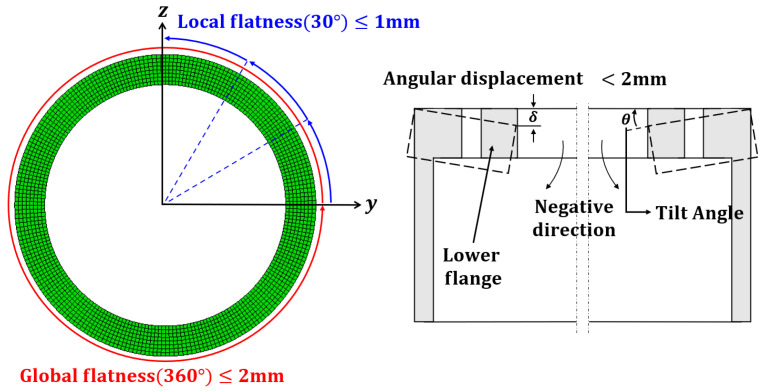
Design requirements for flanges’ flatness and tilt angle.

Deutsches Institut für Bautechnik (DIBt) 2004 provided only qualitative requirements for wind towers [18]. In 2008, flatness tolerance was proposed to complement the qualitative requirements of DIBt owing to the enlargement of wind towers [19]. A tolerance of local flatness sectors (within 30°) of 0.5 mm and global flatness (360°) of 2.0 mm must be satisfied. However, historically, there was no problem when a tolerance of 1.0 mm local flatness was applied. It was determined through experimental research and experience that satisfying the tolerance of local flatness is difficult. In DIBt 2012, the tolerance for the local flatness sector (within 30°) was relaxed to 1.0 mm [20]. An axial direction deformation represents the tilt angle as the slope of the existing and final flange shape. Therefore, the tilt angle can be expressed as the angular displacement. The angular displacement equally changes with the change in flatness, as shown in Figure 2, and a tolerance of 2.0 mm must be satisfied.

## 4. Calculation of Flatness and Tilt Angle of the Flange by Welding

**Figure 3:**
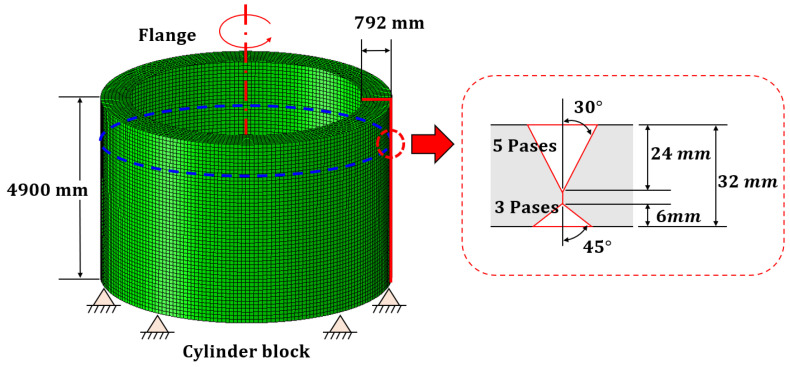
The shape of the wind-tower block, flange, and weld zone.

The research object of this study was a cylindrical block of a 3.6 MW wind tower, with a flange attached for connection between blocks [21]. The flange was connected to the block via butt welding. The block shape and weld zone are shown in Figure 3. The shape of the welded and welding sequence varies depending on the working environment and among them, the representative shape and welding sequence were selected and reflected in the study. A three-dimensional model composed of shell elements was constructed to obtain the deformation due to butt welding of the block and flange; the material used was S355j2 structure steel, and the material properties are shown in Table 1 [17,22,23]

The SDB method was used to obtain the deformation due to butt welding. This method simulates the inherent strain in the member during heating and cooling as internal shrinkage due to welding. Inherent strain refers to strain that is not recovered even when structural restraints or loads are removed. The inherent strain $\left(\epsilon^{*}\right)$ is obtained by subtracting the elastic strain $\left(\epsilon^{e}\right)$ from the total strain $\left(\epsilon^{total}\right)$, as shown in Equation (1), and this strain is equal to the sum of the thermal strain $\left(\epsilon^{th}\right)$, plastic strain $\left(\epsilon^{p}\right)$, and phase- transformation strain $\left(\epsilon^{tr}\right)$ [4]. The inherent strain can be expressed as a virtual thermal expansion coefficient and temperature boundary conditions. The virtual thermal expansion coefficient is calculated from the material thermal expansion coefficient and melting point, and the temperature boundary condition is calculated considering the shape of the heat-affected zone.

(1) $$\epsilon^{*}=\epsilon^{th}+\epsilon^{p}+\epsilon^{tr}=\epsilon^{total}-\epsilon^{e}$$

**Figure 4:**
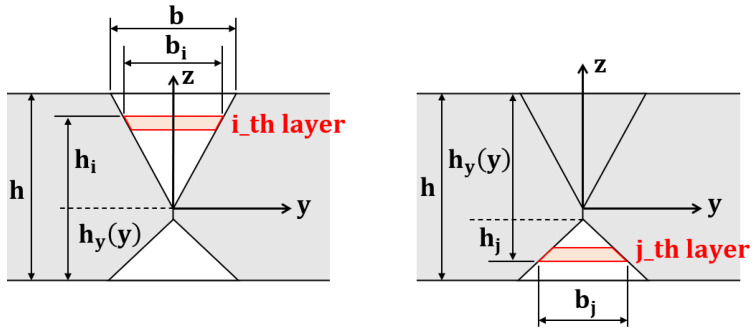
The schematic of the x-groove butt weld modeling.

The butt welding of thick plates is performed by stacking several passes, and the SDB method considering this stacking was proposed by Ha [24,25]. In the proposed method, assuming that each pass has the same cross-sectional area and is stacked in the layer direction, a temperature boundary condition corresponding to the inherent strain was calculated. Butt welding for the X-groove was performed on the upper and lower surfaces of the plate, and the parameters used to obtain the temperature load for each case were defined, as shown in Figure 4. The temperature boundary condition can be calculated using Equation (2) by considering the weld height and width changes. After all the welding passes are performed, the temperature boundary condition corresponding to the inherent strain of the weld can be obtained by superposing all the temperature boundary conditions for each welding pass. This superpose is expressed in Equation (3), and the temperature boundary conditions for the upper and lower surfaces are reversed and superposed.

(2) $$T_{i}=\int_{0}^{b_{i}^{\left(U\right)}}\frac{1}{\left(h_{i}^{\left(U\right)}+h_{y}\left(y\right)\right)\cdot b}\left\{\int_{h_{1}}^{h_{2}}\left(\frac{h_{i}^{\left(U\right)}+h_{y}\left(y\right)}{h}\mp \frac{4z}{h_{i}^{\left(U\right)}+h_{y}\left(y\right)}\cdot \frac{h}{h_{i}^{\left(U\right)}+h_{y}\left(y\right)}\right)dz\right\}dy\;h_{1}=-\frac{h_{i}^{\left(U\right)}+h_{y}\left(y\right)}{2},\;h_{2}=-\frac{h_{i}^{\left(U\right)}+h_{y}\left(y\right)}{2}+\left(h_{i}^{\left(U\right)}+h_{i-1}^{\left(U\right)}\right)$$

where $b_{i}\left(u\right):\;$ Weld width by pass $h_{i}\left(u\right):$ Weld height by pass $h_{y}\left(y\right):$ Lower thickness changes by pass.

(3) $$T=\overset{m}{\underset{i=1}{\sum }}T_{i}+\overset{n}{\underset{j=1}{\sum }}T_{j}$$

where *T_i,j_*: Top and bottom temperature boundary conditions *m*,*n*: Numer of top and bottom passes.

**Figure 5:**
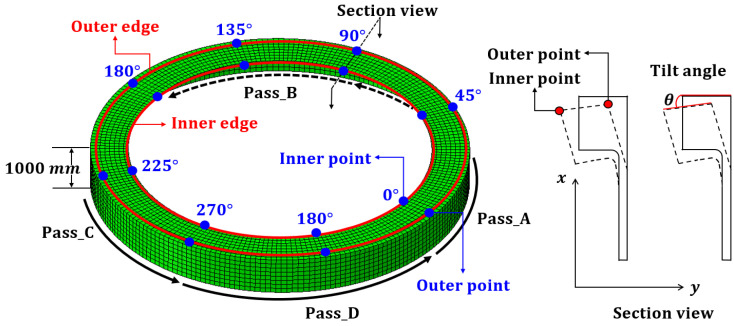
The welding position and sequence, and the measurement position of the block.

**Figure 6:**
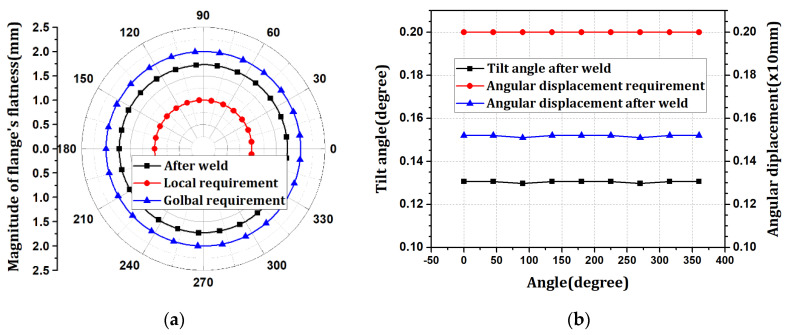
The result of welding procedure: (**a**) flatness changes; (**b**) tilt angle and angular displacement.

The calculated temperature boundary condition was set at the node where the block and flange were joined according to the welding sequence, as shown in Figure 5. It was assumed that all weld shapes and stacking procedures were the same at this time. The block and flange were joined 1000 mm below the top of the flange. The block and flange welding sequence is A > C > B > D, and each welding procedure was performed following completion of the stacking procedure. The measurement positions of the flatness and tilt angle after the welding procedure were completed, shown in Figure 5. The flatness was calculated as the difference between displacements in the x-direction for the inner and outer edges. The tilt angle was calculated using Equation (4) by measuring the inner and outer points’ x- and y-axis displacements. The flatness and tilt angle were calculated at 5/3 and 45° intervals, respectively, as shown in Figure 6, and compared with the design criteria.

(4) $$Tilt\;Angle=\tan^{-1}\left(\frac{\left|x_{i}-x_{o}\right|}{\left|y_{i}-y_{o}\right|}\right)\cdot \left(\frac{180}{\pi }\right),$$

where *x_i_*,*y_i_*: Displacement of inner point for each axis *x_o_*,*y_o_*: Displacement of outer point for each axis.

The flatness was distributed in the range of 1.72096–1.73046 mm in the negative direction. The tilt angle was distributed at 0.129758–0.130618°, with an angular displacement of 1.52 mm. The flatness after welding satisfies the design tolerance of 2.0 mm in the global flatness sector (360°) but not 1.0 mm in the local flatness sector (within 30°). The tilt angle satisfies the design tolerance of 2.0 mm based on the angular displacement. After the welding process, the flange is part of the fastening element for connection with the upper block, and smooth fastening with other blocks is only achieved when the flatness and tilt angle below a certain level are satisfied. Therefore, it is necessary to alleviate the flatness through different processes, such as line heating.

## 5. Calculation of Flange Flatness by Line Heating

Line heating is a process that causes deformation by generating inherent strain owing to the heat, which offsets the previously generated deformation. Line heating moves the input heat source to the desired area with a uniform rule. In addition, knuckles can be prevented at the end of the heating unit by minimizing the length of the remaining portion without heating. If line heating is performed over a constant area, the shape of the HAZ is similar to that of an equilateral trapezoid [5]. The temperature boundary condition can be derived using the SDB method for a given HAZ and can be obtained using Equations (5) and (6).

(5) $$T_{top}=\frac{1}{B\cdot h}\int_{-h/2}^{h/2}b\left(z\right)\cdot \left(1-\frac{4}{h}\cdot z\right)\;dz$$

(6) $$T_{bottom}=\frac{1}{B\cdot h}\int_{-h/2}^{h/2}b\left(z\right)\cdot \left(1+\frac{4}{h}\cdot z\right)\;dz$$

where *B*: Width of the HAZ; *h*: Thickness of the plate; *b*(*z*): Width of HAZ with change in depth *z*.

**Figure 7:**
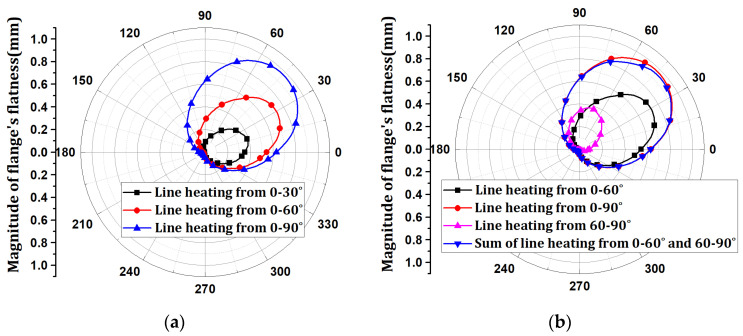
The flatness change by the line heating. (**a**) Flatness changes according to each line heating range; (**b**) Flatness changes according to superposing.

Line heating with a depth of 5 mm and width of 100 mm was performed on the flange. The flange-flatness results according to the line-heating range are shown in Figure 7. When the line heating region was applied in the range of 0–30°, 0–60°, and 0–90° in the radial direction, it was confirmed that the maximum flange flatness increased to 0.38858, 0.72473, and 0.97117 mm, respectively. The range of flatness change gradually increased in proportion to the line-heating range, and flatness changes occurred near the line-heating range. Next, the line heating of 0–60° and 60–90° was superimposed and compared with those of 0–90°. The flatness changes were similar. When the difference in each direction of flatness is expressed as a percentage, the maximum difference is 4.92%, which indicates a considerably similar distribution. Therefore, when line heating is performed at several locations, the flatness result can be obtained by summing those for each line heating.

## 6. Determination of Line Heating Position and Scale Factor Using Optimization

**Figure 8:**
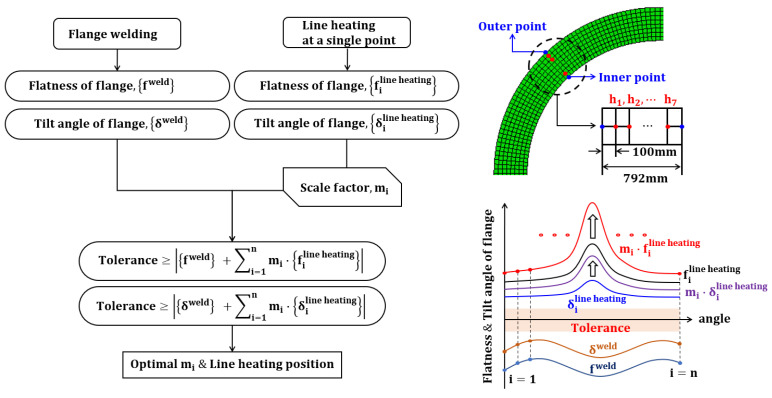
Optimization procedure for determining the optimal line-heating conditions.

The process of deriving the optimal line heating conditions is illustrated in Figure 8. The flatness and tilt angle due to the welding blocks and flanges were organized as angle data. The flatness change and tilt angle due to line heating are organized into angle data for every point between the inner and outer points. The flatness result of line heating can be obtained by superimposing the change due to single-point line heating in the line heating direction, as presented in Section 5. At this time, the flatness change due to each single-point line heating was superimposed in the line heating direction with a scale factor.

The scale factor is a variable that adjusts the magnitude of the line heating, and this adjustment implies that this parameter is related to the magnitude of the flatness. The size of the line heating may vary depending on the line-heating method. However, if constant heating is assumed, the magnitude of the input heat quantity and scale factor are directly proportional. Therefore, the total sum of the scale factors is proportional to the size of the total input heat. By minimizing the scale factor, flatness correction can be performed with minimum input heat. The aforementioned conditions are expressed by Equations (7) and (8). Equation (7) indicates that the sum of the scale factors must have a minimum value as an objective function for optimization. Here, for all single points, the point at which the sum of the scale factors derives the minimum value is selected as the position of line heating. Equation (8) is a constraint indicating that the superposition results of the flatness and tilt angle of welding and line heating must satisfy the design criteria. The design tolerance of flatness was set to 1.0 mm for the local flatness sector (within 30°), and the tilt angle was set to 2.0 mm based on angular displacement. The optimal line heating conditions to satisfy the design criteria were obtained using the optimization conditions.

(7) $$min.\left(\sum_{i=1}^{n}m_{i}\right)$$

(8) $$\left|\left\{f^{weld}\right\}+\sum_{i=1}^{n}m_{i}\cdot \left\{f_{i}^{line\;heating}\right\}\right|\leq Tolerance\;\left|\left\{\delta^{weld}\right\}+\sum_{i=1}^{n}m_{i}\cdot \left\{\delta_{i}^{line\;heating}\right\}\right|\leq Tolerance$$

where *m_i_*: scale factor; *f^weld^*: Flatness of flange by welding; *δ^weld^*: Tilt angle of flange by welding; $f_{i}^{line\;heating}$: Flatness of flange by line heating at a single point; $\delta_{i}^{line\;heating}$: Tilt angle of flange by line heating at a single point

**Figure 9:**
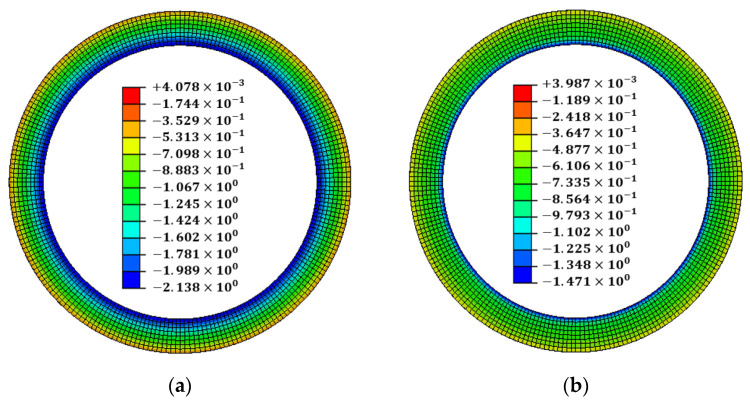
The result of the x-direction deformation: (**a**) after welding; (**b**) after line heating.

**Figure 10:**
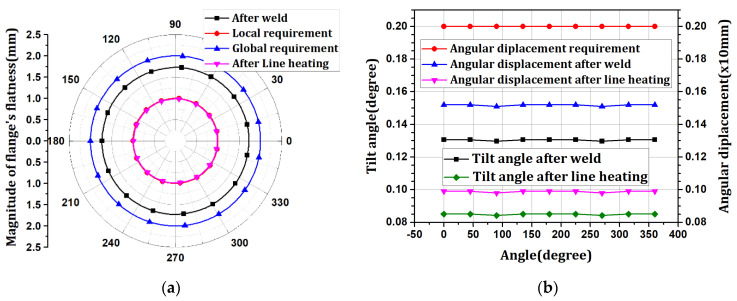
The result of line heating procedure: (**a**) flatness changes; (**b**) tilt angle and angular displacement.

As an optimization result, the scale factor was derived in the range of 0.270677–0.30085 for every point. The line-heating position was determined to be the h6 position, where the sum of the scale factors was minimum. The line-heating direction proceeds in the circumferential direction at the h6 position of 0°. The temperature boundary condition of the line heating was multiplied by the optimization result of the scale factor and applied to the analysis model. When optimal line-heating conditions were applied to the welding analysis results, the maximum flatness decreased from 1.73046 mm to 0.99170 mm. The maximum tilt angle decreased from 0.130618° to 0.085105°, and the maximum angular displacement decreased from 1.52 mm to 0.99 mm. The results of the x-direction deformation of the flange are shown in Figure 9. The flatness and tilt-angle results are shown in Figure 10. It is confirmed that the flatness and tilt angle of the flange satisfy the design tolerance under optimal line heating conditions.

## 7. Verification of Methods for Determining Line Heating Conditions

The size and welding conditions of the wind tower block were varied to verify the proposed method. The size of the wind tower block was divided into 1.5 MW, 3.6 MW, and 5.0 MW. The welding conditions were assumed to be identical for each pass. The flange flatness and tilt angle due to welding and line heating are listed in Table 2. The flatness resulting from welding wind tower blocks and flanges satisfies the design tolerance of global flatness (360°) for all sizes, but not local flatness (within 30°). The optimal scale factor and line heating position were selected by applying the proposed method. Single-point heating locations were divided into h1–h5 (1.5 MW), h1–h7 (3.6 MW), and h1–h7 (5.0 MW) according to the block size order. Line heating was then performed by applying the selected line heating position and scale factor. The flange flatness after line heating satisfied the tolerance of the local flatness (within 30°), and the decrease in the angular displacement was also confirmed according to the decrease in the tilt angle.

**Figure 11:**
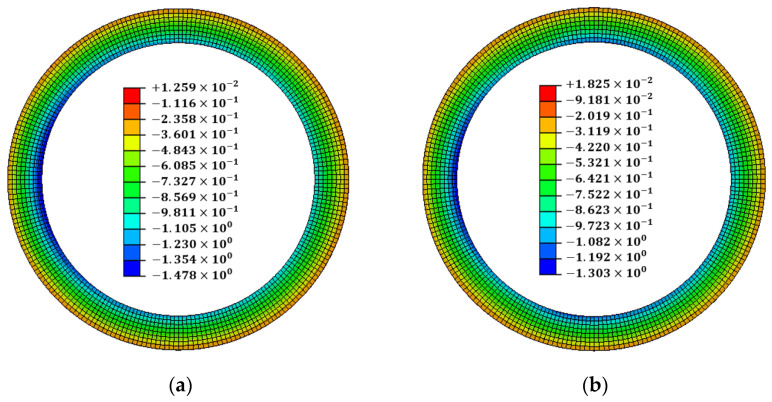
The verification result of the x-direction deformation after the line heating between 120° and 240°: (**a**) after welding; (**b**) after line heating.

**Figure 12:**
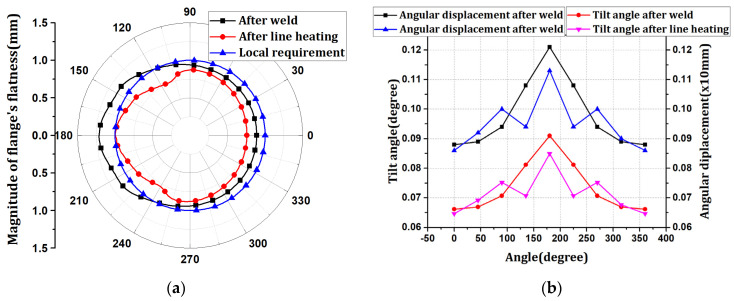
The verification result of the line heating procedure: (**a**) flatness changes; (**b**) tilt angle and angular displacement.

The welding conditions of the wind tower block and flange depend on the working environment and operator. Changes in welding conditions affect the flange flatness. Therefore, welding was performed by changing the conditions for each pass of the 3.6 MW wind tower block and flange. The optimal conditions for line heating were determined using the proposed method. Thus, it was confirmed that if the difference in flatness was large, the scale factor at the location with high flatness increased. Additionally, it was confirmed that the scale factor of the position that satisfied the flatness tolerance was zero. The position at which the scale factor is zero represents where line heating is unnecessary. Therefore, the proposed method can perform line heating by dividing the section for the position at which the deviation of the line heating is large. The results of the x-direction deformation of the flange are shown in Figure 11. The flatness and tilt-angle results are shown in Figure 12.

The welding flatness change occurred at a maximum of 1.21252 mm between 120° and 240°, and line heating was performed as described in this section. In the proposed method, line heating was performed at position h7, and the maximum flatness was reduced to 0.99697 mm. The tilt angle of the flange decreased from 0.090981 to 0.084967°. Simultaneously with the tilt-angle change, the angular displacement decreased from 1.21 mm to 1.13 mm. It was confirmed that the deformation decreased in the section where the line heating was performed. However, the tilt angle and angular displacement increased and then decreased in the place where the line heating was not performed because the deformation was superposed.

## 8. Conclusions

The assembly of the wind tower block is bolted, and the flatness affects the installation tolerance in the bolting process. In order to control flatness in the manufacturing stage, milling and line heating are performed in the field. Among these methods, line heating is performed by the operator’s empirical judgment without clear standards. There are many studies on line heating, but the studies need to suggest clear standards. Therefore, this study proposed a method to determine the optimal line-heating conditions to control the flatness generated by welding a wind-tower block and flange.

The scale factor for line heating that offsets welding deformation was determined using the deformation superposition method and optimization. The proposed method was verified by changing the size and welding conditions of the wind tower. The size of the wind tower is 1.5 MW, 3.6 MW, and 5.0 MW; the welding conditions are the same. Scale factors for size were derived in the range 0.022865–0.032911, 0.270677–0.30085, and 0.116164–0.18979 at positions h4, h6, and h7, respectively. Welding conditions were changed for each pass at 3.6 MW. The scale factor for the change in welding conditions was in the range of 0.159787–0.771813 at the h7 position and was derived from the position that did not satisfy the flatness tolerance. It was confirmed that the flatness satisfies all the design tolerances when the scale factor for size and welding condition change is superimposed with the temperature boundary condition of line heating.

The scale factor is a variable that adjusts the size of the line heating. Adjusting the line heating size means that the flatness level can be changed. Using these scale factors, the size and location of the line heating are determined; based on this, the width and height of the line heating and the starting position can be suggested. In addition, line heating may be performed by dividing the position where the scale factor is zero according to the deviation of the flatness. Therefore, this can be used for cross-, lattice-, and point-heating.

## Figures and Tables

**Table 1:** Material properties of S355J2 structure steel.

Property	S355J2
Young’s Modulus, Mpa	210
Density, kg/m^3^	7850
Poisson’s ratio	0.3
Tensile strength, Mpa	630
Yield strength, Mpa	355
Melting point, °C	1530

**Table 2:** The verification result for the block-size and welding condition changes.

Design Criteria					
Procedure	Block Size	Flatness (mm)	Tilt Angle (°)	Angular Displacement (mm)	Heating Location
Welding	1.5_MW	1.20992–1.21582	0.117766–0.117861	1.41	
	3.6_MW	1.72096–1.73046	0.129758–0.130618	1.51–1.52	
	5.0_MW	1.51567–1.51682	0.109238–0.109239	1.51	
Line heating	1.5_MW	0.99446–0.99899	0.098562–0.099393	1.18–1.19	h4
	3.6_MW	0.98234–0.99170	0.084246–0.085105	0.98–0.99	h6
	5.0_MW	0.94640–0.94805	0.068737–0.068738	0.95	h7

## Data Availability

The data presented in this study are available on request from the corresponding author.
